# Anthropogenically enhanced chemical weathering and carbon evasion in the Yangtze Basin

**DOI:** 10.1038/srep11941

**Published:** 2015-07-07

**Authors:** Jingheng Guo, Fushun Wang, Rolf David Vogt, Yuhang Zhang, Cong-Qiang Liu

**Affiliations:** 1 College of Resources and Environmental Sciences, China Agricultural University, Beijing 100193, China; 2 School of Environmental and Chemical Engineering, Shanghai University, Shanghai 200433, China; 3 Department of Chemistry, University of Oslo, 0315 Oslo, Norway; 4 State Key Laboratory of Environmental Geochemistry, Institute of Geochemistry, Chinese Academy of Sciences, Guiyang 55002, China

## Abstract

Chemical weathering is a fundamental geochemical process regulating the atmosphere-land-ocean fluxes and earth’s climate. It is under natural conditions driven primarily by weak carbonic acid that originates from atmosphere CO_2_ or soil respiration. Chemical weathering is therefore assumed as positively coupled with its CO_2_ consumption in contemporary geochemistry. Strong acids (i.e. sulfuric- and nitric acid) from anthropogenic sources have been found to influence the weathering rate and CO_2_ consumption, but their integrated effects remain absent in the world largest river basins. By interpreting the water chemistry and overall proton budget in the Yangtze Basin, we found that anthropogenic acidification had enhanced the chemical weathering by 40% during the past three decades, leading to an increase of 30% in solute discharged to the ocean. Moreover, substitution of carbonic acid by strong acids increased inorganic carbon evasion, offsetting 30% of the CO_2_ consumption by carbonic weathering. Our assessments show that anthropogenic loadings of sulfuric and nitrogen compounds accelerate chemical weathering but lower its CO_2_ sequestration. These findings have significant relevance to improving our contemporary global biogeochemical budgets.

Dissolution of silicate and carbonate minerals by external protons (i.e. chemical weathering) mobilizes chemical mineral components to the ocean through inland water continuum. Under natural conditions, carbonic acid from atmospheric CO_2_ or soil respiration is the dominant proton donor. Subsequent carbonate deposition in ocean leads to CO_2_ sequestration for different time scale^1^. In contemporary geochemistry, this chemical weathering, positively coupled with atmospheric CO_2_ consumption, is generally presumed to be in a long-term quasi-steady state since pre-industrial times^2^,^3^. On the other hand, recent studies in small catchments have found that the loadings of anthropogenic strong acids alter weathering rates and CO_2_ uptake by supplementing or substituting the weak carbonic acid^4^,^5^,^6^,^7^. However, their integrated evaluation is still challenging in the world largest river basins, due to significant spatial heterogeneities in operation mechanisms that vary essentially with the proton sources/fates and rock composition. Such a conundrum hampers to evaluate their significance in global biogeochemical budget.

The Yangtze River is one of the largest rivers in the world, with a drainage area of 1.8 × 10^6^ km^2^ (see Supplementary Information and Supplementary Figure S1 online). Silicate and carbonate rocks are interlaced in the basin, with the coverage of ca. 56% and 44%, respectively. Strong weathering of carbonate rocks generates among the largest discharge of solutes and CO_2_ consumption of the world large rivers^2^,^3^,^8^. Since the last 80s, the Yangtze Basin has become the most developed region in China. Inherent anthropogenic emissions of sulfur and nitrogen pollutants have resulted in continuous increases in sulfate and nitrate in the Yangtze River, indicating an anthropogenic acidification in the basin^9^,^10^.

The geochemical effects of anthropogenic acid loadings on chemical weathering, solute transport and inorganic carbon fluxes are evaluated by integrating several data-sets spanning the temporal and spatial variations in water chemistry of the Yangtze River. The main objectives of this study are (1) to identify the main mechanisms governing mobilization and transport of base cations and inorganic carbon to the river, based on regional water chemistry data during past and present periods, (2) to assess the contribution of sewage ammonium emission to river acidification and bicarbonate protonation, using temporary and spatial water quality data, and (3) to quantify the integrated impact of anthropogenic acid loading on weathering rates and inorganic carbon fluxes, by interpreting the overall proton budget in the Yangtze Basin.

## Results

### Terrestrial acidification and chemical weathering

**Figure 1:**
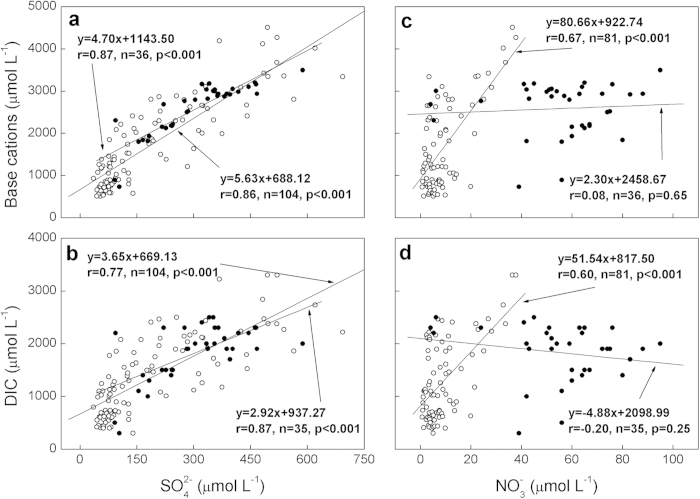
Spatial correlations of base cations and DIC with SO_4_^2−^ and NO_3_^−^ in the Yangtze River. **a**, base cations versus SO_4_^2−^; **b**, DIC versus SO_4_^2−^; **c**, base cations versus NO_3_^−^; **d**, DIC versus NO_3_^−^. Past data (1958–1980) are average values at each hydrologic station (open circles). Recent data (filled black circles) are from a regional survey conducted in 2006. Base cations were calculated as the charge sum of Ca^2+^, Mg^2+^, K^+^ and Na^+^. DIC for past data is represented as bicarbonate (HCO_3_^−^), which is essentially equal to DIC in the pH ranges of the Yangtze River.

Sulfuric acid loading promotes mineral dissolution and thereby leaching of solutes to river^4^,^5^,^11^,^12^,^13^,^14^. As shown in Fig. 1a, the concentrations of base cations (i.e. equivalence sum of Ca^2+^, Mg^2+^, K^+^ and Na^+^) are found to correlate (*p* < *0.001*) with sulfate concentrations in both past (1958–1980) and recent data (2006), confirming the contributions of sulfuric acid to mineral dissolution. Monovalent cations (K^+^ + Na^+^) response much less than divalent cations (Ca^2+^ + Mg^2+^) to sulfate increase (see Supplementary Figure S2 online). This indicates that sulfuric weathering is principally the same on both minerals, but that the effect is stronger on the carbonates. Sedimentary paleocarbon is released when carbonate minerals are dissolved by sulfuric acid, enhancing the DIC delivery to watercourse. Since sulfuric weathering does not consume atmospheric CO_2_ the sulfate induced increase in flux of DIC (Fig. 1b) should be attributed entirely to the dissolution of sedimentary paleocarbon in carbonate minerals. A geochemical assessment of strontium (Sr) data, from a regional survey of the Yangtze River^14^, further substantiates that this DIC increase is due to enhanced carbonate rock weathering by sulfuric acid (see Supplementary Information and Supplementary Figure S3 online).

Nitric acid plays a similar role as sulfuric acid in mineral weathering^6^,^7^,^15^. Prior to the 1990s the dominant source for nitrate in the Yangtze River was agricultural fertilizer^10^. Protons produced through nitrification in the soils are consumed *in situ* by the dissolution of soil minerals. There were therefore positive correlations between NO_3_^−^, base cations and DIC in the river during the past period (1958–1980) (Fig. 1c,d). Enhanced nitrogen fertilizer has accelerated soil acidification since the 1980s^16^, so stronger nitric weathering is conceptually expected. However, there is no significant relationship between nitrate, base cations and DIC in recent (2006) data. This apparent discrepancy is mainly due to the shift in the main nitrate sources from diffuse agricultural runoff of nitrate to sewage drainage of ammonium. In-stream nitrification of sewage ammonium has thereby become another important source of nitrate to the Yangtze River during the current period^17^. An important distinction here is that this nitric acid is not involved in terrestrial weathering.

### Aquatic acidification and DIC evasion

**Figure 2:**
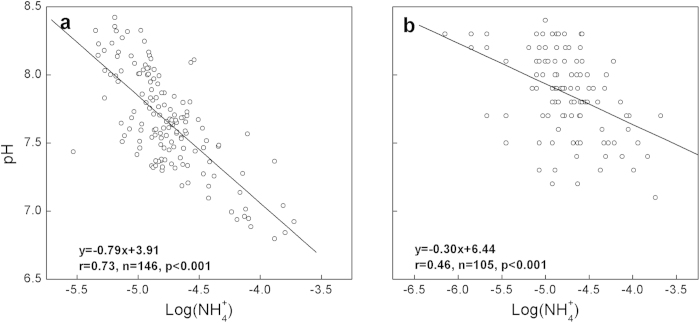
Spatial correlations between annual average pH and log(NH_4_^+^). **a**, annual averaged values at 19 key cross sections. **b**, annually averaged values for 105 regular cross sections. pH and log(NH_4_^+^) were calculated based on averaged molar concentrations of H^+^ and NH_4_^+^, respectively.

At present, there is about 6.77 × 10^5^ ton ammonium nitrogen (NH_4_^+^-N) discharged annually into the Yangtze River^18^. Its nitrification releases proton directly to the water, and therefore causes acidification. However, water acidification by this large proton source is not observed directly by studying long-term pH trends (see Supplementary Information and Supplementary Figure S4 online). Instead, we document it here using two regional data sets from the Chinese Ministry of Environment Protection (MEP). Annual average pH significantly (*p* < *0.001*) decreases with increased log of NH_4_^+^ concentration at 19 key sections (Fig. 2a). Extending the assessment to additional 105 sections, thereby covering more regional variations, confirms the similar relationship (Fig. 2b). Acidification through sewage ammonium nitrification is thus postulated to be a major factor governing regional differences in the water quality. This hypothesis is also strengthened by a separate statistical analysis (see Supplementary Information and Supplementary Figure S5 online).

Assuming that all the sewage ammonium is nitrified, releasing acidity to river, the corresponding CO_2_ outgasing would be 1.16 Tg C yr^−1^. However the actual acidification induced CO_2_ efflux is likely considerably larger than this, since nitrification in watercourse is not the only proton source to the Yangtze River. A surplus of strong acids (i.e. not consumed by terrestrial weathering) contributes to increase the outgassing of DIC both in soils and surface waters. Especially in watersheds devoid of carbonate minerals more protons are consumed through bicarbonate protonation due to the lower solubility of silicate minerals. In some upper catchments of the Yangtze Basin receiving acid deposition, stream water is extremely acidic (pH ranging from 4 to 5)^19^. In this pH range, practically all the DIC exists as CO_2_ and is liable to be released to the atmosphere. Residual acidity entering the Yangtze River will be further consumed by DIC alkalinity, enhancing riverine CO_2_ evasion (see Supplementary Information and Supplementary Figure S6 and S7 online).

### Flux budgets for the Yangtze Basin

**Figure 3:**
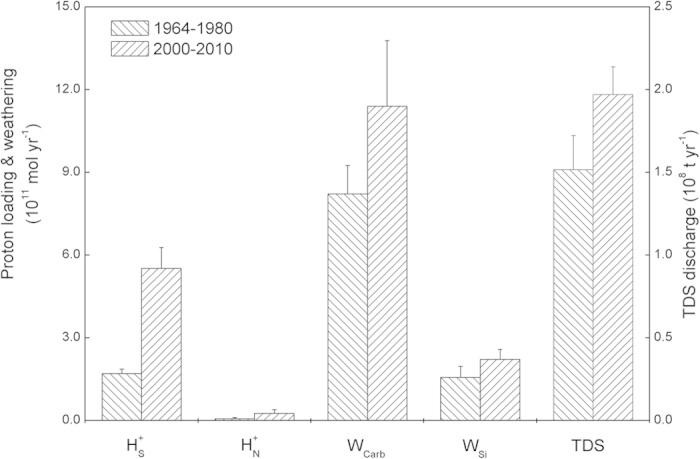
Proton loading, chemical weathering rates and discharge of Total Dissolved Salt from the Yangtze Basin during past and present periods. H^+^_S_ and H^+^_N_ denote the proton flux from sulfur and nitrogen processes, respectively. W_Carb_ and W_Si_ represent the proton flux consumed through chemical weathering of carbonate and silicate minerals, respectively. TDS donates total dissolved salt. All fluxes are statistically significant different between the two periods (*p* < *0.001*).

Proton loadings to the Yangtze Basin were calculated for two time periods (i.e. 1964–1980 and 2000–2010) based on measured discharge fluxes of sulfate and nitrate to the ocean (Fig. 3). During the past three decades, sulfuric proton input increased by 2.2 times (*p* < *0.001*), from 1.70 × 10^11^ to 5.52 × 10^11^ mol yr^−1^. Acid rain has been conceived as the major cause for this sulfate increase, since the Yangtze Basin is mainly located within the Chinese acid rain region^9^. However, recent studies indicate that about 60% of sulfate in the Yangtze River originates from sulfide (e.g. pyrites) oxidation^11^,^14^. Sulfide oxidation is promoted by geological explorations through exposing sulfide gangue to aerobic conditions (i.e. acid mining drainage)^5^,^20^. The Yangtze Basin is the major production region of mining resources (e.g. coal and various metals) in China^21^. The increasing exploration activities are therefore partly accounting for the enhanced sulfuric acid inputs. Though nitric acid loading is less than sulfuric acid, it increased by 3.3 times (*p* < *0.001*) during the same period. Agricultural fertilizer and urban sewage are proved as the two major sources for this increased reactive nitrogen in the Yangtze River^17^,^18^.

The anthropogenic loading of strong mineral acids has augmented (*p* < *0.001*) the rock weathering within the Yangtze Basin and thereby increased the flux of solutes discharged into the ocean (Fig. 3). Discharge of total dissolved salt (TDS) increased by 30% during the past 30 years, from 1.52 × 10^8^ to 1.97 × 10^8^ ton yr^−1^. Carbonate flux from weathering increased by 38.7%, from 8.22 × 10^11^ to 11.4 × 10^11^ mol yr^−1^. Even though the weathering of silicates is less than that of carbonate minerals, its relative increase was 42%. Bicarbonate percentage of total anionic charge decreased from 85% in historical period to 66.9% in present times. This is basically accounted for by the increase in sulfate (from 9% to 25%). Molar concentration ratios of divalent cations (Ca^2+^ + Mg^2+^) over bicarbonate increased (*p* < *0.001*) from 0.51 ± 0.10 to 0.68 ± 0.13. The historic value is close to the theoretical one (i.e. 0.5) for carbonic carbonate weathering, while the present value clearly deviates from this value, documenting a significant anthropogenic influence.

To evaluate the influence of acid loading on inorganic carbon fluxes, the DIC budgets during the past and present in the Yangtze Basin were compared (Table 1). During 1964–1980, carbonic weathering consumed 10.81 Tg C CO_2_ per year, 90% of which is accounted for by carbonate weathering. The discharge to ocean was 20.75 Tg C, basically equal to the DIC input by chemical weathering. This budget confirms the common assumption that inland river system serves only as a passive conveyer pipe for DIC under natural conditions^22^. Anthropogenic acids have increased the paleocarbon mobilization from 0.64 in the past to the current 4.47 Tg C yr^−1^, accounting for 48% of natural flux (i.e. 9.25 Tg C yr^−1^ in Table 1). However, this increase is not accounted for by an elevated DIC discharge, implying an annual DIC loss of 3.79 Tg. A proton budget shows that 2.09 ± 0.22 Tg C is released annually to the atmosphere as CO_2_ due to carbonate buffering. The remaining 1.70 ± 0.15 Tg C yr^−1^ is assumed assimilated as organic carbon, which may subsequently be released to atmosphere as CO_2_ again by respiration. Global estimates show that 67 to 76% of terrestrially derived organic carbon is evaded as CO_2_ from inland water, estuary and inshore ocean^23^,^24^,^25^,^26^,^27^. This implies that between 1.04 and 1.41 Tg of the DIC derived from organic carbon is released as CO_2_ through respiration. Total DIC outgassing may then amount to 3.28 Tg C yr^−1^ from the Yangtze Basin, accounting for 30% of total CO_2_ consumption by chemical weathering. The remaining 24 to 33% of the organic fixated carbon is stored in sediments of lakes, reservoirs, estuaries and ocean^24^,^25^,^26^,^27^, corresponding to 0.51 Tg C yr^−1^ in this case (Table 1). According to global estimates the sediments in lakes and reservoirs along the Yangtze River may account for 0.32 (0.19–0.41) Tg C yr^−1^ of the sedimented organic carbon (i.e. 12%–22%).

### Conceptual overview

**Figure 4:**
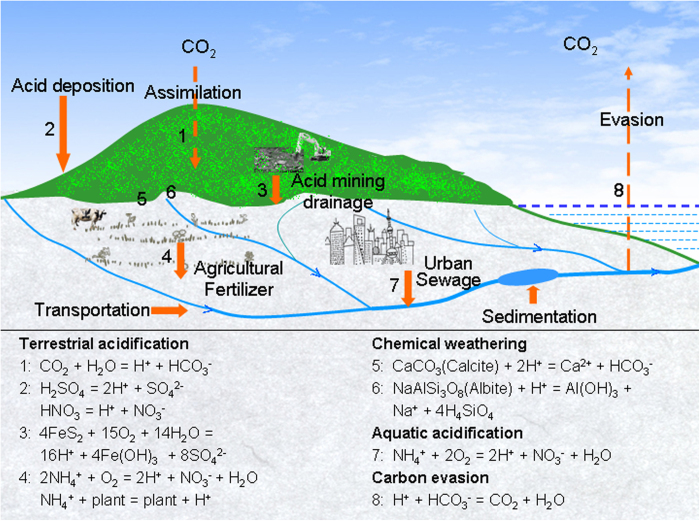
Graphical abstract illustrating the influence of anthropogenic acid loadings on chemical weathering and inorganic carbon processes. Calcite and albite exemplify carbonate and silicate minerals, respectively. This figure was drawn by software Adobe Photoshop 7.0.

Overall, anthropogenic proton loadings enhance chemical weathering but offset its CO_2_ consumption in the Yangtze Basin (Fig. 4). However, the mechanisms and their effects conceptually vary with rock types: (A) In regions covered by carbonate rocks the protons from atmospheric deposition, acid mining drainages and agricultural fertilizers accelerate dissolution of carbonate minerals, and increase the flux of their dissolved solutes (mainly as Ca^2+^ and HCO_3_^−^) to the watercourse (2, 3, 4 and 5 in Fig. 4). During their transportation to ocean, some of the bicarbonate (HCO_3_^−^) is released as CO_2_ due to in-river proton production by sewage nitrification (7 and 8 in Fig. 4). Part of the bicarbonate is fixated into organic carbon through photosynthesis by aquatic photosynthesis. This organic carbon is either evaded as CO_2_ by respiration or stored in sediments; (B) In regions devoid of carbonate rocks only a part of the anthropogenic proton loading (2, 3 and 4 in Fig. 4) is consumed by silicate weathering (6 in Fig. 4) due to their lower solubility. The remaining acidities instead protonate the bicarbonate derived from carbonic weathering (1 and 6 in Fig. 4). Furthermore, some anthropogenic acidity is leached from acid soil as H^+^ and acidic Al, and subsequently evades river DIC together with the acidity from nitrification (7 and 8 in Fig. 4). Since both carbonate and silicate rocks are widely distributed in the Yangtze Basin their integrated influence is in fact more convoluted than outlined by this generic description.

## Discussions

Globally, anthropogenic acidification has become a widespread environmental challenge induced mainly by acid rain^20^,^28^,^29^, agricultural nitrogen fertilizer^15^,^16^,^22^, urban sewage, and acid mining drainage^5^,^20^,^30^. These anthropogenic proton sources are disrupting the natural couplings between chemical weathering and atmospheric CO_2_ consumption, constituting important disturbances to the fundamental biogeochemical processes in the atmosphere, land and ocean. Substitution of strong acids to weak carbonic acid enhances the evasion of inorganic carbon to atmosphere, contributing to the riverine CO_2_ outgassing^31^ and offsetting the CO_2_ sequestration by carbonic weathering. Moreover, supplementation of strong acids to carbonic acid accelerates chemical land erosion and increases thereby the solute discharge^7^,^12^,^14^,^32^. Similarly, agricultural liming, widely used to mitigate soil acidification, also increases ion leaching^33^,^34^. Finally, changes in riverine discharge chemistry, both in terms of mass concentration and stoichiometry, may be altering the element biogeochemistry in ocean. For example, sulfuric weathering of carbonates will lead to transient CO_2_ emission from ocean in geological time scale^11^,^13^.

## Methods

### Data sources

Regional water chemistry (i.e. major ions) of the Yangtze River is assessed in regards to past and present conditions. Past chemistry were obtained by compiling monitoring data from hydrological year books (1958–1980)^35^, while data describing more recent conditions are from a synoptic regional study^14^. The past data are based on a very comprehensive dataset of water chemistry at 105 cross sections along the river. Average data (n = 10–225) over the monitoring period were used for each site since data series at most of sections were discontinuous. These data, with the exception of nitrate, were previously published by Chen *et al.* (2002)^9^. The data were recompiled in order to incorporate NO_3_^−^ data, required for the current discussion. Recent monitoring data of water chemistry for Yangtze River are scarce due to discontinuation of hydrological year books in the late 1980s. A precious data set from a regional survey in 2006^14^ is instead used as a measure for the current chemistry of the Yangtze River.

Discharge fluxes of solutes to the ocean (i.e. the East China Sea) from the Yangtze Basin were calculated based on water chemistry and hydrological data at Datong hydrological station. This station is located at tidal limit region of the Yangtze River. Past hydrological fluxes were compiled from the hydrological year books^35^. Past discharge fluxes (1964–1980) of major ions were set as averages (n = 17) of their annual discharge fluxes. Historical water discharge flux and DIC flux at Datong were also previously published by Wang *et al.* (2007)^36^. Recent (2001–2010) average (n = 5) discharge fluxes of chemical constituents were calculated based on literature values for water chemistry and corresponding water discharge at Datong station^14^,^37^,^38^,^39^.

Water quality data (i.e. pH, NH_4_^+^-N, Dissolved oxygen (DO) and Chemical Oxygen Demand (COD) or Biochemical Oxygen Demand at 5 days incubation (BOD_5_)) were compiled from the datasets generated by a monitoring program organized by the Chinese Ministry of Environment Protection (MEP). This program monitored the water quality for 21 years (1991–2011) at two cross sections, Zhutuo and Jiujiang. The Zhutuo section is located directly upstream of Chongqing municipality, while Jiujiang section is situated downstream of the metropolis, close to Jiujiang city, Jiangxi province. Two additional datasets, covering the spatial variation in water quality, are based on monitoring data from 19 key and 105 regular sections (Figure S1). Weekly water quality data (including COD, not BOD_5_) since 2004 at the 19 key cross sections were downloaded from the MEP website (http://datacenter.mep.gov.cn/report/getCountGraph.do?type=runQianWater). Annual average water quality data for year 2010 at the 105 regular sections (including BOD_5_, instead of COD) were compiled from a MEP report^40^. All sampling and analysis were conducted according to MEP occupational standards.

### Calculation methods

Changes in water pH, dissolved CO_2_ (H_2_CO_3_^*^) and CO_2_ partial pressure (P_CO2_) were theoretically estimated according to CO_2_-H_2_O equilibriums in a closed system, where external acid loadings decrease alkalinity without changes in total dissolved inorganic carbon (DIC) (*eq. S1–S6*). In these calculations four acid loading levels (50, 100, 150 and 200 μmol L^−1^ H^+^) were assessed, which cover the conceivable span in acid loadings to the Yangtze River. The range of DIC studied was from 500 to 2 500 μmol L^−1^, spanning the current water chemistry of the Yangtze River.

Proton (i.e. H^+^) budget calculations were conducted based on the assumption that all external acid inputs were consumed through chemical weathering and loss of river alkalinity within the basin. This is sound, since high bicarbonate concentrations at Datong station effectively buffer any increase in proton concentration in the river. All external proton loadings were assumed to originate from carbonic acid, sulfuric acid, and nitrogen compounds. Carbonic acid flux was estimated as the CO_2_ consumption by chemical weathering. All sulfate discharged to the sea is presumed to have originally been sulfuric acid derived from acid deposition, sulfide oxidation and so on. A minor contribution from dissolution of evaporites (e.g. gypsum) was disregarded as it would have no net influence on the final proton budget. Nitrogen compounds have different contributions to the proton loading; the overall acidification potential of nitrogen processes in the Yangtze Basin was thus estimated using a previously published method (*eq. S8*).

The major proton consumption was through chemical weathering. Weathering rates in the basin, and thereby their proton consumption, were deduced from the base cation discharges to the ocean. Each divalent base cation (i.e. Ca^2+^ and Mg^2+^) released through carbonate weathering conceptually consumes 1.0 equivalent proton (*eq. S9*), while silicate weathering uses 2.0 equivalent proton (*eq. S11*). Literature studies indicate that silicate weathering only contributes ca. 4.8% to total release of divalent base cation on the basin scale^2^,^14^. Carbonate weathering (W_Carb_) was therefore set as the divalent base cation flux at Datong station, after subtracting the contribution from silicate weathering (*eq. S10*). Monovalent base cations (Na^+^ + K^+^) mainly originate from silicate mineral weathering where H^+^ consumption is equal to their mole concentration (*eq. S12*). The flux of monovalent base cations was calculated from the riverine discharge after correction for the contribution from dissolution of evaporates and sea salt deposition, using chloride as tracer. Silicate weathering (W_Si_) was expressed as the proton consumption by (Ca^2+^ + Mg^2+^) and (Na^+^ + K^+^) released from silicate minerals (*eq. S13*). The remaining important proton depletion mechanism is the protonation of bicarbonate. The amount of protons consumed by protonation of bicarbonate was thus approximated as the difference between external proton input and proton consumption by chemical weathering (*eq. S14*).

Basin budget for DIC was obtained through assessing the difference between the fluxes of its input sources and output sinks (*eq. S15*). In these calculations two sources of DIC were considered. One was atmospheric CO_2_ (Carbon_Atm_) captured during carbonic weathering. The other was sedimentary paleocarbon in carbonate minerals, mobilized by natural carbonic acid (Paleocarbon_Nat_) and anthropogenic strong acids (Paleocarbon_Ant_). Delivered DIC was either discharged directly to the ocean or processed in the river. The former was calculated based on DIC fluxes at Datong station, while the latter flux was estimated as the difference between DIC input and riverine discharge. There are two pathways for the riverine processed DIC. One is CO_2_ outgassing due to protonation of bicarbonate, which was set as equal to the amount of protons consumed by bicarbonate in the proton budget. The other is fixation into organic carbon through photosynthesis. Most of this terrestrially derived organic carbon will be oxidized to CO_2_ again in river, estuary and coastal regions within one year^23^,^24^,^25^,^26^,^27^; therefore the net assimilation of DIC (i.e. ‘Assimilation’ in *eq. S15*) is considered as rather small.

Detail rationales for above calculations can be found in the Supplementary Information.

## Additional Information

**How to cite this article**: Guo, J. *et al.* Anthropogenically enhanced chemical weathering and carbon evasion in the Yangtze Basin. *Sci. Rep.* **5**, 11941; doi: 10.1038/srep11941 (2015).

## Supplementary Material

Supplementary Information

## Figures and Tables

**Table 1:** Budget of dissolved inorganic carbon (DIC, in Tg C yr^−1^) in the Yangtze Basin.

Periods	Input^1^			Discharge	River processed^2^		
	Carbon_Atm_	Paleocarbon_Nat_	Paleocarbon_Ant_		Total	Outgassing	Assimilation
1964–1980	10.81 ± 0.12	9.25 ± 0.14	0.64 ± 0.08	20.75 ± 3.18	−0.06	/	/
2000–2010	10.81 ± 0.12	9.25 ± 0.14	4.47 ± 0.54	20.73 ± 3.52	3.79 ± 0.61	3.28(3.08–3.53)	0.51(0.37–0.61)

^1^Carbon_Atm_ is the atmospheric CO_2_ uptake flux by chemical weathering. Paleocarbon_Nat_ and Paleocarbon_Ant_ denote the sedimentary paleocarbon inputs from natural and anthropogenic carbonate weathering, respectively.

^2^River processed fluxes are calculated as the difference between inputs and discharges for the two periods. Outgassing and assimilation for current period (2000–2010) is estimated based on the acidity budgets in the Yangtze Basin. Negative value for river processed carbon means a minor positive contribution by DOC respiration to riverine DIC, indicating no net fluxes by outgassing or assimilation of DIC from weathering.
