# Supplementary material for: Anthropogenically enhanced chemical weathering and carbon evasion in the Yangtze Basin
Source: Sci Rep. 2015 Jul 7;5:11941. doi: 10.1038/srep11941 (PMC4493640; doi:10.1038/srep11941)
Supplement: Supplementary Information [file srep11941-s1.pdf]

Supplementary Information *for*

Anthropogenically enhanced chemical weathering and carbon evasion in the Yangtze Basin

Authors: Jingheng Guo[1*], Fushun Wang[2], Rolf David Vogt[3], Yuhang Zhang[1], Cong-Qiang Liu[4*]

[*]Corresponding author. E-mail: guojingheng@cau.edu.cn (J. G.); liucongqiang@vip.skleg.cn (C.-Q. L.)

Contents

1. Supplementary Materials and Methods
   1.1. The Yangtze Basin
   1.2. DIC equilibrium calculation
   1.3. Proton budget for the Yangtze Basin
      1.3.1. Proton loadings
      1.3.2. Chemical weathering and proton consumption
      1.3.3. Proton budget
   1.4. DIC budget calculation
2. Supplementary Text
   2.1. Terrestrial acidification and chemical weathering
   2.2. Aquatic acidification and DIC outgassing
3. Supplementary References
4. Supplementary Figures

1. Supplementary Materials and Methods

1.1. The Yangtze Basin

The Yangtze River (or Changjiang in Chinese) is the longest river in Asia, and one of the largest river systems in the world. It originates from the Qinghai-Tibet Plateau and flows 6 400 km eastward to the East China Sea. Its drainage area covers 1.8×10^6 km^2, comprising about 1/5 of Chinese land area. The Yangtze Basin is located in the subtropical monsoon climate zone, with an average annual precipitation of 1 100 mm. Spread throughout the basin are carbonaceous sedimentary minerals (limestone, sandstone and shale), comprising about half (44%) of the land area[2]. The chemistry in the Yangtze River is thus governed by carbonate rock weathering, with HCO_3^- and Ca^{2+} dominating the major ion composition[9,14]. Coal and ore deposits, rich in sulfides (e.g. pyrite), interbedded in the sedimentary rocks are rather common in the Yangtze Basin[9,14,21].

The Yangtze Basin is the most important industrial region in China, generating 40% of the total national industrial production value. Agricultural land comprises 14% of the watershed, providing 40% of China's cereal production. Since the middle of last century, the basin has been subject to significant anthropogenic pollution, with acid rain being a regional environmental problem since the 1970s[9,41]. Furthermore, widespread acid mining drainage has contributed significantly to the total acid loading through oxidation of sulfides to sulfuric acid in gangue and ore slag deposits[21]. Total loading of reactive nitrogen has also increased in the basin during the past 30 years, mainly due to nitrogen fertilization and increased emissions of NOx from fuel burning[42]. Concurrently, agricultural soils in the Yangtze Basin have become acidified due to overuse of nitrogen fertilizer[16]. Ammonium (NH_4^+) has become a major pollutant in Yangtze River along with a rapid growth in urbanization along the river. Data from the Chinese national pollutant census state that 6.77×10^5 t NH_4-N, mainly from domestic sewage, is discharged annually into the Yangtze River and its tributaries[18].

1.2. DIC equilibrium calculation

Theoretical calculations of pH, dissolved CO_2 ($H_2CO_3^*$) and CO_2 partial pressure (P_{CO_2}) were conducted according to CO_2-H_2O equilibriums (*eq. S1-S4*) in a closed system[43,44]. Alkalinity, being a proxy for the equivalent sum of proton acceptors (i.e. bases of weak acids), is basically conceived as a measure of the equivalent sum of bicarbonate (HCO_3^-) and carbonate (CO_3^{2-}) concentrations. In low DIC rivers the contribution by other proton acceptors (e.g. organic anions, A^- in eq. S5) may account for large parts of titrated alkalinity. Likewise, the measured alkalinity derived from changing the pH from sample pH to pH 4.5 (i.e. < 31.5 μeq/L) may constitute a significant part of the measured alkalinity in low DIC systems. DIC concentrations in the Yangtze River are significantly higher than the global median value for rivers, due to the predominance of carbonate minerals[9]. Bicarbonate (HCO_3^-) and carbonate (CO_3^{2-}) are therefore the dominant contributors to water alkalinity (Alk) (*eq. S5*), so that titrated alkalinities (to pH 4.5) may be set equal to the equivalent sum of HCO_3^- and CO_3^{2-} concentrations (*eq. S5*)[14]. The relationship between alkalinity and DIC can thus be expressed by *eq. S6*. At any given [DIC] and [Alk], the pH, P_{CO_2}, [$H_2CO_3^*$], [HCO_3^-] and [CO_3^{2-}] can be calculated using *eqs. S1* to *S3*, after obtaining the equivalent fraction coefficient (α) from *eq. S6*.

$$CO_2(g) + H_2O \Leftrightarrow H_2CO_3^*(aq); \frac{[H_2CO_3^*]}{p_{CO_2}} = K_H \qquad (eq.\ S1)$$

$$H_2CO_3^*(aq.) \Leftrightarrow H^+ + HCO_3^-; \frac{[H^+]\times[HCO_3^-]}{[H_2CO_3^*]} = K_1 \qquad (eq.\ S2)$$

$$HCO_3^- \Leftrightarrow H^+ + CO_3^{2-}; \frac{[H^+]\times[CO_3^{2-}]}{[HCO_3^-]} = K_2 \qquad (eq.\ S3)$$

$$[DIC] = [H_2CO_3^*(aq.)] + [HCO_3^-] + [CO_3^{2-}] \qquad (eq.\ S4)$$

$$[Alk] = [HCO_3^-] + 2[CO_3^{2-}] + [A^-] + [OH^-] - [H^+] \approx [HCO_3^-] + 2[CO_3^{2-}] \qquad (eq.\ S5)$$

$$[Alk] = [HCO_3^-] + 2\times[CO_3^{2-}] = [DIC]\times\frac{K_1\times[H^+] + 2\times K_1\times K_2}{[H^+]^2 + K_1\times[H^+] + K_1\times K_2} = [DIC]\times\alpha \qquad (eq.\ S6)$$

External acid loading decreases [Alk] and α in *eq. S6*. This inherently increases the [$H_2CO_3^*$(aq)]

and thus the partial pressure of CO_2 (P_{CO_2}) in the closed system. Where this CO_2 supersaturated water is exposed to open air, the CO_2 will diffuse to atmosphere reducing the difference in P_{CO_2}. As a starting point for the theoretical calculation, we set acid loading as zero where [Alk] equals to [DIC]. This equivalence point is corresponding to the equilibrium of $CaCO_3$ dissolution by CO_2, i.e. where chemical weathering of carbonate mineral is not influenced by anthropogenic strong acids (e.g. H_2SO_4 and HNO_3).

The nitrification of 6.77×10^5 t NH_4^+-N discharged to Yangtze River, with an annual water discharge of 9.51×10^{11} m^3, corresponds to an acidification of ca. 100 μmol L^{-1} H^+, assuming that all of the NH_4^+ is nitrified (*eq. S7*). Since ammonium nitrification is not the only proton source to Yangtze River, two additional acid loadings (50 and 200 μmol L^{-1} H^+) were assessed in order to cover the conceivable span in acid loading. The DIC range was studied from 500 to 2 500 μmol L^{-1}, spanning the current water chemistry of Yangtze River.

$$NH_4^+ + 2O_2 \Rightarrow NO_3^- + 2H^+ + H_2O \qquad (eq.S7)$$

1.3. Proton budget for the Yangtze Basin

1.3.1. Proton loadings

It is assumed that all external proton loading is from carbonic acid (H^+_C) and sulfuric acid (H^+_S), or derived from nitrogen compounds (H^+_N). Carbonic acid (H^+_C) was estimated as the atmospheric CO_2 consumption flux by weathering, based on that aquatic CO_2 ($H_2CO_3^*$) deprotonates according to *eq. S2* and *S3* during chemical weathering. In this study the value used for CO_2 consumption in the Yangtze Basin was the average of compiled values found in literature, and assumed as constant for both past and present periods[2,3, 14,38]. All sulfate discharged to the sea at Datong station is presumed to have originally been sulfuric acid (H^+_S) derived from acid deposition, sulfide oxidation and so on. This is disregarding a minor contribution from dissolution of evaporites (e.g. gypsum). Field surveys indicate that the contribution from evaporites to the sulfate flux is insignificant, though good quantitative data are

lacking[2,9,14]. Furthermore, dissolution of sulfate from evaporites inherently leads to an equivalent flux of base cations, which is included in the measure for chemical weathering. This possible minor overestimation of acid loading due to evaporate dissolution will thus not influence the estimation of acidity budget.

Nitrogen processes have different contributions to the proton loading, with organic N mineralization, NH_4^+ assimilation, NH_4^+ nitrification and NO_3^- assimilation generating 1 equivalent H^+ loss, 1 equivalent H^+ gain, 2 equivalent H^+ gains and 1 equivalent H^+ loss, respectively[16,45,46]. The overall acidification potential of nitrogen processes in the Yangtze Basin can be expressed by *eq. S8*. The amount of nitrate in sewage is low and in China mainly urea and manure are applied as nitrogen fertilizers[16]. The nitrate input ($NO_3^-{}_{In}$) to the river was therefore assumed to be negligible and set to zero. The annual ammonium input ($NH_4^+{}_{In}$) was set equal to the input from sewage discharge (i.e. 4.83 $\times 10^4$ mol). Discharged fluxes of nitrate and ammonium at Datong hydrological station were used for nitrate ($NO_3^-{}_{Out}$) and ammonium output ($NH_4^+{}_{out}$) in *eq. S8*.

$$H^+{}_N = (NH_4^+{}_{In} - NH_4^+{}_{Out}) + (NO_3^-{}_{Out} - NO_3^-{}_{In}) \qquad (eq.\ S8)$$

1.3.2. Chemical weathering

Weathering of carbonate and silicate minerals consumes external protons and releases base cations through runoff. Total chemical weathering rates (expressed as their proton consumption) in the basin may therefore be deduced from the base cation discharge fluxes. However, the origin of base cations (i.e. carbonate or silicate mineral) need be distinguished in such deductions, since the equivalence ratios between base cation release and proton consumption are different between carbonate and silicate weathering (eq. S9, S11 and S12). For carbonate weathering (W_{Carb}), a confounding challenge is that the divalent base cations in discharge ($(Ca^{2+}+Mg^{2+})_{Dis}$) originate from both silicate and carbonate minerals. Therefore, divalent base cations from carbonate weathering ($(Ca^{2+}+Mg^{2+})_{Carb}$) is calculated according to *eq. S10*, with β denoting the contribution from silicate weathering. Literature studies

indicate that silicate weathering contributes ca. 4.8% to total release of divalent base cations in the basin[2,14]. The release of a divalent base cation through carbonate weathering ((Ca^{2+}+Mg^{2+})$_{Carb}$) conceptually consumes 1.0 equivalent H^+ (*eq. S9*). Carbonate weathering (W_{Carb}) is therefore estimated according to *eq. S10*, setting β to 4.8%. Minor contribution of (Ca^{2+}+Mg^{2+}) flux by evaporate dissolution is not subtracted, thereby counterbalancing the erroneous contribution by evaporites to sulfuric acidity input, as stated above.

$$CaCO_3(Calcite) + H^+ \Rightarrow Ca^{2+} + HCO_3^- \quad \text{(eq. S9)}$$

$$W_{Carb} = (Ca^{2+} + Mg^{2+})_{Carb} = (1-\beta)(Ca^{2+} + Mg^{2+})_{Dis} \quad \text{(eq. S10)}$$

$$CaAl_2Si_2O_8(Anorthite) + 2H^+ + 6H_2O \Rightarrow 2Al(OH)_3 + Ca^{2+} + 2H_4SiO_4 \quad \text{(eq. S11)}$$

$$NaAlSi_3O_8(Albite) + H^+ + 7H_2O \Rightarrow Al(OH)_3 + Na^+ + 3H_4SiO_4 \quad \text{(eq. S12)}$$

$$W_{Si} = (Na^+ + K^+)_{Si} + 2(Ca^{2+} + Mg^{2+})_{Si} = (Na^+ + K^+)_{Si} + 2\beta(Ca^{2+} + Mg^{2+})_{Dis} \quad \text{(eq. S13)}$$

Weathering of silicate minerals release both monovalent (Na^++K^+) and divalent cations (Ca^{2+}+Mg^{2+}). Monovalent base cation flux from silicate weathering ((Na^++K^+)$_{Si}$) consumes 1 equivalent H^+ (*eq. S12*). This loading is calculated from the riverine discharge after correction for the contribution from dissolution of evaporates and sea salt deposition, using chloride as tracer. Each divalent cation from silicate weathering ((Ca^{2+}+Mg^{2+})$_{Si}$) consumes 2 equivalent H^+ due to the formation of non-ionic ortho-silicic acid (*eq. S11*). The proton consumption by silicate mineral weathering (W_{Si}) is thus expressed by *eq. S13*.

1.3.3. Proton budget

It is assumed that all acid inputs are consumed through chemical weathering and alkalinity loss (-△Alk) within the basin. This is sound since the pH at Datong hydrological station remains stable and high through strong buffering by high bicarbonate concentrations. The acidity budget of the Yangtze Basin can thus be expressed by *eq. S14*.

$$H^{+}{}_{C} + H^{+}{}_{S} + H^{+}{}_{N} = W_{Si} + W_{Carb} - \Delta Alk. \qquad (eq.\ S14)$$

1.4. DIC budget calculation

The input-output budget of DIC is expressed by *eq. S15*. Weathering releases DIC to the Yangtze River through three mechanisms. Carbonic weathering of carbonate and silicate minerals captures the atmospheric CO_2 ($Carbon_{Atm}$) constituting the H^{+}_{C} flux expressed in *eq. S14*. Carbonic weathering of carbonate minerals (*eq. S16*) mobilizes an equivalent amount of atmospheric carbon and lithogenic paleocarbon ($Paleocarbon_{Nat}$) to the river. When carbonates instead are dissolved by anthropogenic strong acids (e.g. H_2SO_4 and HNO_3), the paleocarbon released to river ($Paleocarbon_{Ant}$) is equal to the mole concentration of divalent cations (i.e. Ca^{2+} and Mg^{2+}) (*eq. S17, S18*). Therefore, $Paleocarbon_{Ant}$ values were set as the difference between (Ca^{2+}+Mg^{2+}) released from overall carbonate weathering (i.e. $(Ca^{2+}+Mg^{2+})_{Carb}$ in *eq. S10*) and from carbonic carbonate weathering. The latter value is equal to the CO_2 consumption by carbonate minerals that was set as the average of literature values[2,3,14,38].

$$Carbon_{Atm} + Paleocarbon_{Nat} + Paleocarbon_{Ant} = Discharge + Outgas + Assimilation \qquad (eq.\ S15)$$

$$CaCO_3 + CO_2 + H_2O = Ca^{2+} + 2HCO_3^{-} \qquad (eq.\ S16)$$

$$2CaCO_3 + H_2SO_4 = 2Ca^{2+} + 2HCO_3^{-} + SO_4^{2-} \qquad (eq.\ S17)$$

$$CaCO_3 + HNO_3 = Ca^{2+} + HCO_3^{-} + NO_3^{-} \qquad (eq.\ S18)$$

Discharge in *eq. S15* denotes the DIC flux delivered to the sea, and was set equal to the efflux at Datong hydrological station. The difference between inputs and discharge is defined as the river processed DIC. This flux includes the CO_2 outgassing and fixation by aquatic primary producers through photosynthesis. Outgassing of riverine DIC occurs through two mechanisms: The major process is protonization of bicarbonate due to the proton loading and dehydration of carbonic acid, with a quantity that is equal to the reduction in inorganic carbon alkalinity (i.e. -△Alk in *eq. S14*). The minor one is from the oxidation of DOC. Global estimates show that about 70% of the DOC is oxidized

to CO_2 in inland watercourses, estuaries and coastal regions[23-27]. The rest of the DOC is either precipitated in lakes, reservoirs, estuaries, or ocean and is defined as 'Assimilation' in *eq. S15*.

2. Supplementary Text

2.1. Terrestrial acidification and chemical weathering

Chemical weathering of silicate and carbonate minerals is the major process for consumption of external proton loadings to terrestrial systems. Its major products eventually are delivered to the ocean through inland watercourses. River chemistry is thus commonly used in contemporary geochemical studies as an important indicator of chemical weathering within watersheds[2,3]. Base cation concentrations are seen to increase significantly with sulfate and nitrate levels, except for nitrate in recent (2006) data (Figure S2). The regression slopes for divalent cations (Ca^{2+}+Mg^{2+}) are much steeper than those for monovalent cations (K^{+}+Na^{+}), indicating considerably slower weathering rates of silicate minerals than for carbonates. This statistically derived result is consistent with more quantitative estimations found in literature[2,3,14]. We argue here that terrestrial acidification in the Yangtze Basin accelerates chemical weathering and thereby increases the flux of solutes to the watercourse. During recent years, nitrification of sewage ammonium has become an additional major source to nitrate in the Yangtze River[17,18]. This nitrification process releases proton directly to the river, without contribution to terrestrial chemical weathering. This is the cause for lack of any clear relationships between overall nitrate and base cation concentrations in the recent (2006) data (Figure S2c and S2d).

Contributions of carbonate weathering to river chemistry can also be evaluated using the strontium (Sr) concentration and its isotope ratio. Weathering of carbonates is characterized by higher Sr concentration and lower $^{87}Sr/^{86}Sr$ ratios, while silicate weathering leads to relatively lower Sr concentration with higher $^{87}Sr/^{86}Sr$ ratios[47,48]. Sr concentrations and $^{87}Sr/^{86}Sr$ ratios in the Yangtze River are found to be significantly positively and negatively correlated, respectively, with sulfate

concentration (Figure S3a and S3b). Nitric acid has similar effect on carbonate weathering as sulfuric acid[6,15], though the in-river processing of ammonium to nitrate causes there to be no significant relations between nitrate and Sr geochemical data. Still, the terrestrial neutralization of sulfuric and nitric acids by especially carbonate weathering (*eq. S17*) increases the loading of bicarbonates to the Yangtze River. This causes the Sr concentration and $^{87}Sr/^{86}Sr$ ratio to also be correlated with DIC (Figure S3c and S3d). In contrast to carbonic weathering (*eq. S16*), carbonate dissolution by strong acids (*eq. S17, S18*) releases bicarbonate derived only from sedimentary paleocarbon in the carbonate minerals.

2.2. Aquatic acidification and DIC outgassing

There are two major sources for external acid loading to the Yangtze River. In sub-catchments devoid of carbonates, residual acidity (mainly as Al^{3+} and H^+) is leached from the soils and delivered to the upper streams. Some headwater streams in the Yangtze Basin are therefore acidic or even extremely acidic[19,41]. During recent years, ammonium discharged along with sewage has become a major cause for pollution in the Yangtze Rive[17]. Its nitrification (*eq. S8*) releases protons directly into the water, and is thus devoid of terrestrial acid neutralization. Instead this acid loading serves to acidify the Yangtze River.

At Zhutuo cross section, upstream of Chongqing, the water pH is generally found to decrease along with the increase in NH_4^+-N concentration over time during the past two decades (1991 to 2011) (Figure S4a). A negative co-variation (r=0.16, n=251, p=0.01) between pH and ammonium is also observed at Jiujiang cross section, downstream of Jiujiang city, though there is no clear time trend (Figure S4b). The lack of a clear pH decline with time at Jiujiang section is nevertheless not implying a lack of river acidification, because river acidification cannot be assessed simply by assessing temporary pH trends. Principle component analysis (PCA) was instead used to assess the underlying co-variations between H^+, NH_4^+, dissolved oxygen (DO) and COD/BOD (Figure S5). Figure S5a and S5b show

inter-parameter relationship in regards to temporary co-variations in the monitoring data for the past two decades at Zhutuo and Jiujiang section, respectively. Ammonium (NH_4^+) and H^+ show positive loading while DO was negatively loaded along the first component (PC1). This loading pattern indicates that NH_4^+ increases the H^+ production while decreases (i.e. consuming) DO in river, which complies conceptually with the nitrification process (*eq. S8*). This PC1s explain 45.6 and 47.0% of the temporal variations in the measured parameters at Zhutuo and Jiujiang sites, respectively. Nitrification and resulting acid loading are therefore assessed to be the major components governing the water quality changes during past 21 years. Parameter loading plots from PCA of the regional water quality variations at the 19 key and 105 regular cross sections are shown in Figure S5c and S5d, respectively. The loading plots *a* to *d* in Figure S5 appear generally similar. Acidification due to ammonium nitrification is therefore claimed to be the main governing mechanism for both temporal and spatial variations in these water quality parameters. In other words, the ammonium discharged with sewage is acidifying the Yangtze River.

Even though the river has been suffering from continuous acidification, substantial pH drops can not be expected in the Yangtze River. This is due to the fact that chemical weathering, especially carbonate weathering, delivers alkalinity to the river which efficiently is consuming the external acid loadings. Theoretical calculations demonstrate that nearly all of the total acid loading is consumed by protonation of dissolved carbonates, giving rise to a negligible proton (H^+) increase (Figure S6). The results indicate therefore instead that the external acid input is mainly consumed by protonation of bicarbonate, enhancing CO_2 evasion as the system is open to the atmosphere. At any given DIC level, acid loadings elevate the CO_2 partial pressure (P_{CO_2}, along vertical axis of Figure S7), leading to enhanced CO_2 evasion. Furthermore, at any given acid loading P_{CO_2} increases with the DIC (along horizontal axis of Figure S7). This implies that the increase in DIC flux resulting from land acidification, as argued above, is augmenting the river outgassing of CO_2. Within a conceivable range

of acid (i.e. 50-200 μmol L^{-1}) and DIC (500-2 500 μmol L^{-1}) loading, there remains enough residual alkalinity to buffer river pH, keeping it stable. We therefore conclude that aqueous acidification in the Yangtze River accelerates the gas evasion of inorganic carbon, causing a significant release of CO_2 to the atmosphere, instead of causing a decline in pH.

3. Supplementary References

41. Larssen, T., *et al.* Acid deposition and its effects in China: An overview. *Environ. Sci. Policy* **2**, 9-24 (1999).
42. Liu, X. J., *et al*. Enhanced nitrogen deposition over China. *Nature* **494**, 459-462 (2013).
43. Appelo, C. J. A., Postma, D. *Geochemistry, groundwater and pollution.* (A.A. Balkema, Rotterdam, 1999).
44. Stumm, W., Morgan, J. J. *Aquatic chemistry: chemical equilibria and rates in natural water*. (John Wiley & Sons, ed. 3, NewYork, 1996).
45. Reuss, J. O., Johnson, D. W. *Acid deposition and the acidification of soils and waters*. (Springer-Verlag, NY, 1986).
46. van Breemen, N., Mulder, J., Driscoll, C. T. Acidification and alkalinization of soils. *Plant Soil* **75**, 283-308 (1983).
47. Palmer, M. R., Edmond, J. M. Controls over the strontium isotope composition of river water. *Geochim. Cosmochim. Acta* **56**, 2099-2111 (1992).
48. Wadleigh, M. A., Verizer, J., Brooks, C. Strontium and its isotopes in Canadian rivers: Fluxes and global implications. *Geochim. Cosmochim. Acta* **49**, 1727-1136 (1985).

4. Supplementary Figures

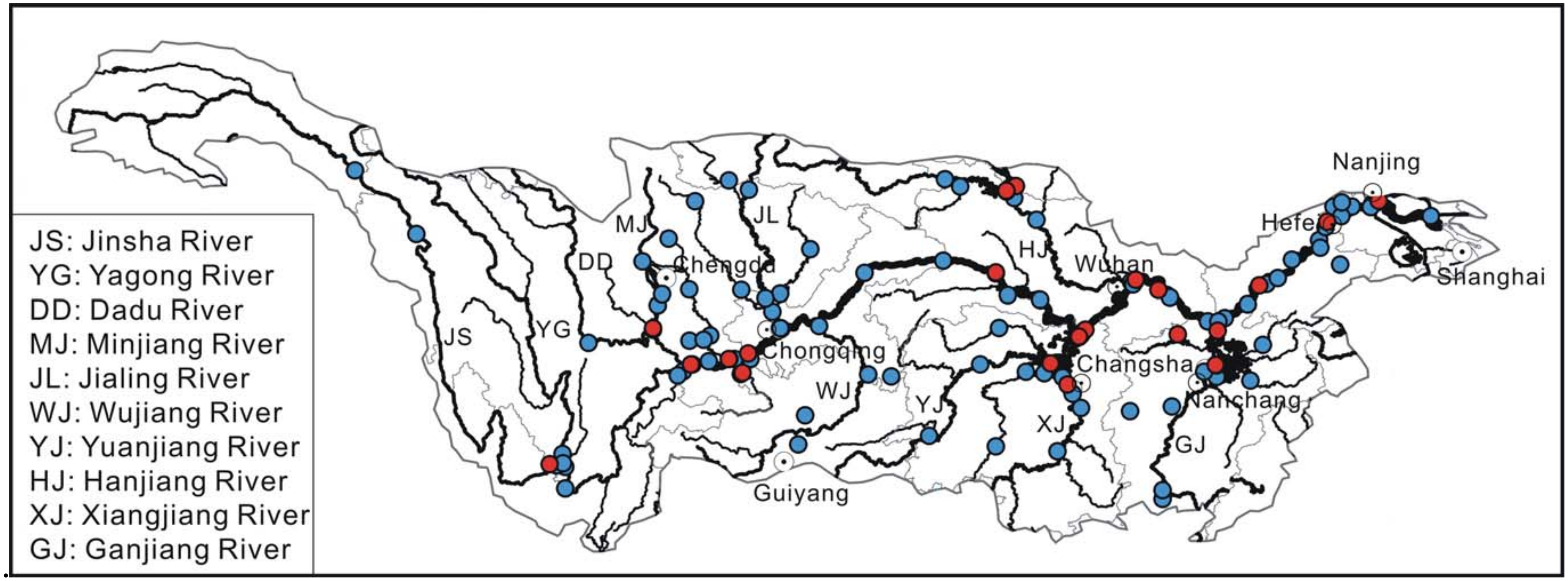

Figure S1 │ Geographical distribution of MEP monitoring cross sections in the Yangtze River Basin. Red and blue dots represent key and regular sections, respectively. This map was drawn by software ArcGis 9.3.

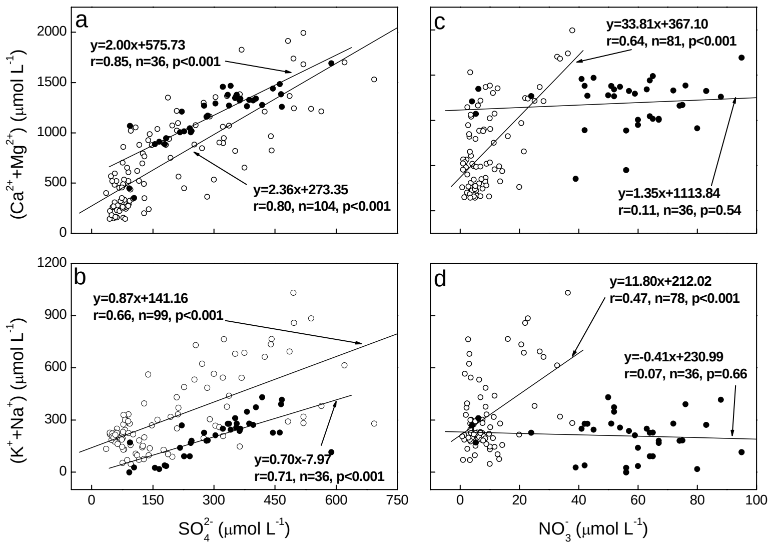

Figure S2 │ Spatial correlations of base cations with SO_4^{2-} and NO_3^- in the Yangtze River. a, (Ca^{2+}+Mg^{2+}) versus SO_4^{2-}; **b**, (Na^++K^+) versus SO_4^{2-}; **c**, (Ca^{2+}+Mg^{2+}) versus NO_3^-; **d**, (Na^++K^+) versus NO_3^-. Past data (1958-1980) are average values at each hydrologic station (open circles) (Supplementary Information). Recent data (filled black circles) are from a regional survey conducted in 2006.

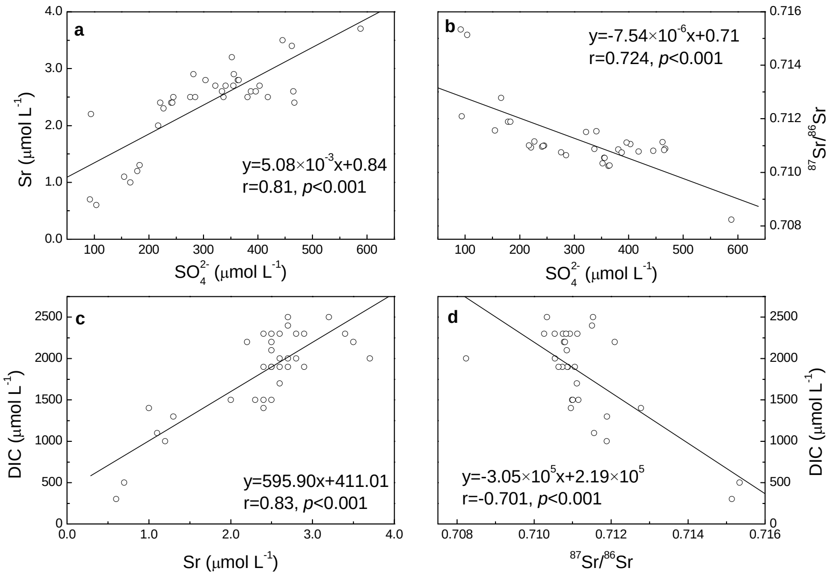

Figure S3 │ Relationships of strontium (Sr) and $^{87}Sr/^{86}Sr$ ratio with SO_4^{2-} and DIC in Yangtze River. a, Sr versus SO_4^{2-}; **b**, $^{87}Sr/^{86}Sr$ ratio versus SO_4^{2-}; **c**, DIC versus Sr; **d**, DIC versus $^{87}Sr/^{86}Sr$ ratio. Data were compiled from Chetelat *et al.* (2008)[14].

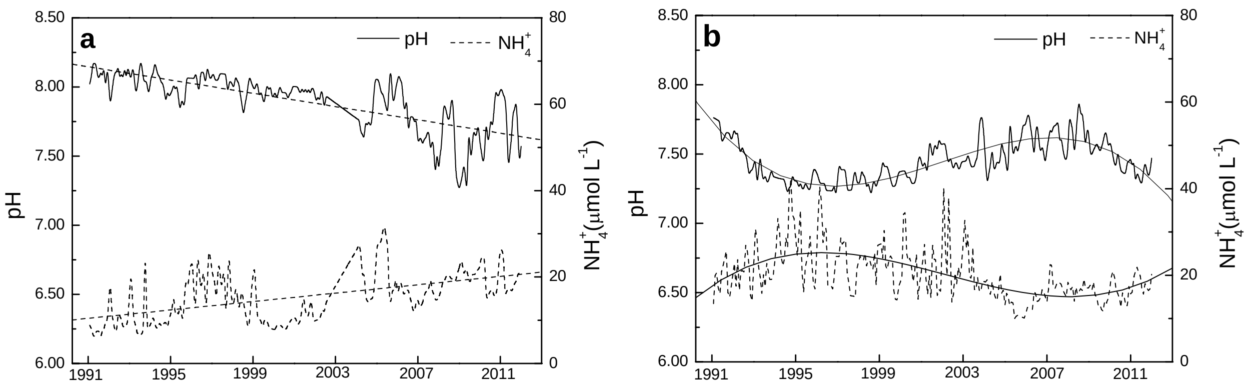

Figure S4 │ Temporal variations in pH and NH_4^+ at two cross sections during the past two decades. a, Zhutuo section near Chongqing (upstream); **b**, Jiujiang section in Jiangxi province (downstream).

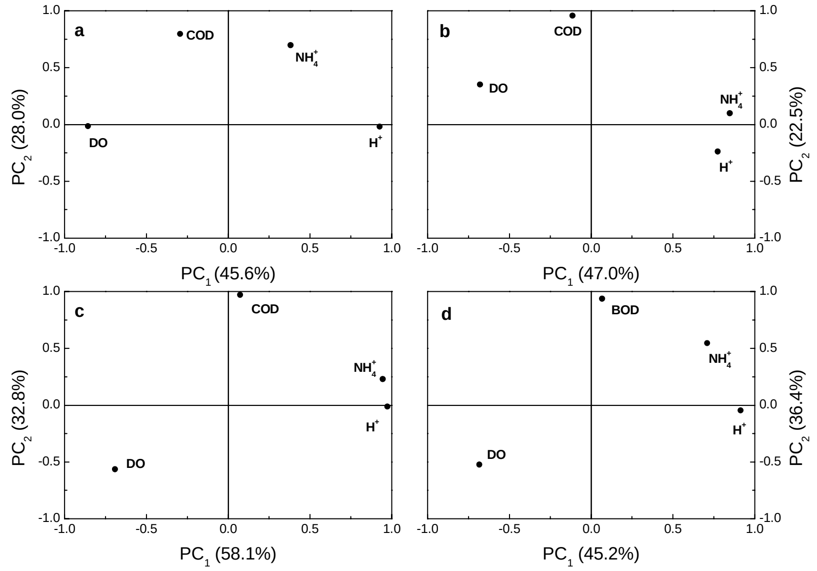

Figure S5 │ PCA loading plots of NH_4^+, H^+, DO and COD/BOD_5. a and **b**, temporal (1991-2011) variation at Zhutuo and Jiujiang cross section, respectively. **c** and **d**, spatial variation at 19 key and 105 regular cross sections, respectively.

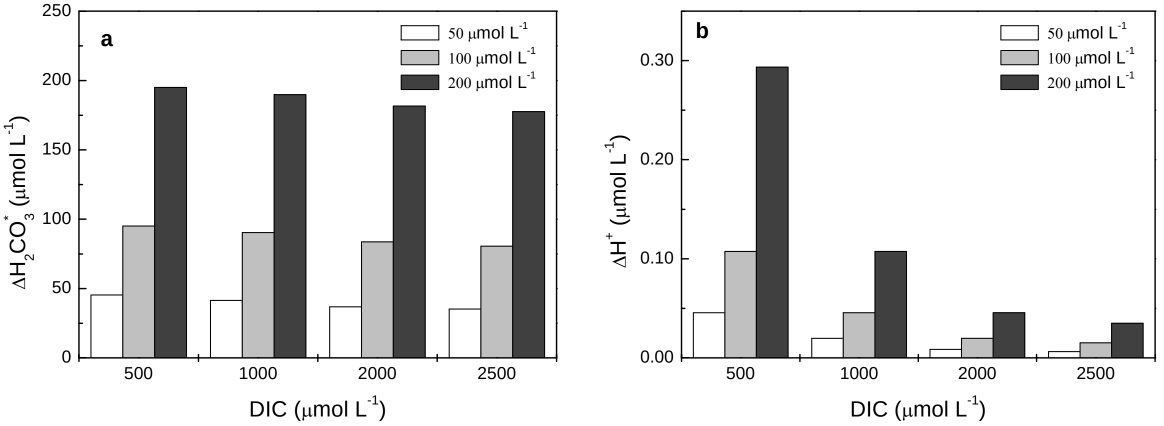

Figure S6 │ Changes in dissolved CO_2 ($\Delta H_2CO_3^*$) and protons (ΔH^+) at different DIC and external acid loadings. a, increase in dissolved CO_2 ($\Delta H_2CO_3^*$); **b**, proton increase (ΔH^+).

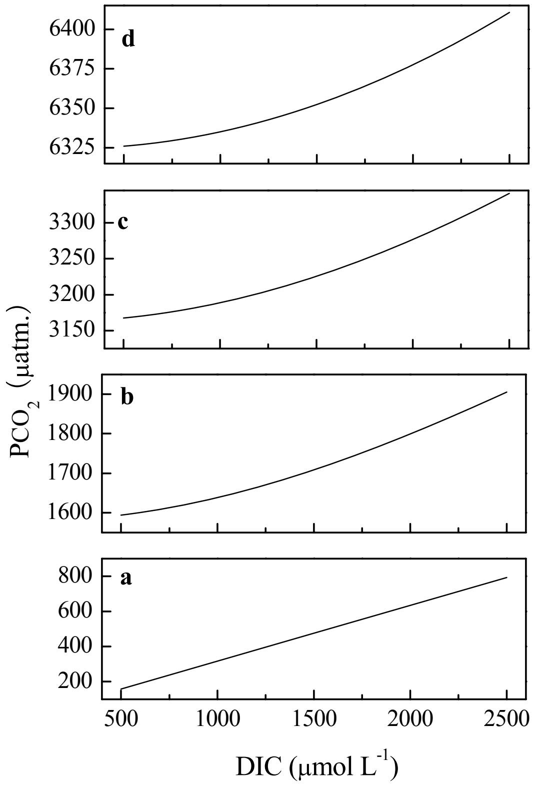

Figure S7 │ Theoretical calculations of changes in partial pressure of CO_2 (P_{CO_2}) under different acidity (H^+) loadings. a, 0 μmol L^{-1}; **b**, 50 μmol L^{-1}; **c**, 100 μmol L^{-1}; **d**, 200 μmol L^{-1}.
